# Supplementary material for: MetaRibo-Seq measures translation in microbiomes
Source: Nat Commun. 2020 Jun 29;11:3268. doi: 10.1038/s41467-020-17081-z (PMC7324362; doi:10.1038/s41467-020-17081-z)
Supplement: Supplementary file 10 — Supplementary Data 7 [file 41467_2020_17081_MOESM10_ESM.zip › File2/Confidence_VeryHigh_Taxonomy/332678_out.krona.html]

Javascript must be enabled to view this page.

members
magnitude
magnitudeUnassigned
count
unassigned
taxon
rank

332678\_out

118

2
superkingdom
118

976
phylum
118

118
200643
class

118
order
171549

11
815
family

11
genus

SRS074964\_contig\_number\_4558
816
1

817

SRS018313\_contig\_number\_7298SRS024388\_contig\_number\_9845
species
2

1
species

SRS1041095\_contig\_number\_1328
821


SRS015431\_contig\_number\_15442
246787
species
1

28116

SRS053573\_contig\_number\_2791
species
1

2
species
469589

SRS023971\_contig\_number\_450SRS1041092\_contig\_number\_3362

1
species
310298

SRS014459\_contig\_number\_9145

162156

SRS077127\_contig\_number\_10832
species
1

species

SRS056519\_contig\_number\_1314
818
1

1
family
2005519

genus
1348911
1

1
species

SRS144506\_contig\_number\_35616
1099853

1
family
171552

1
genus
838

species

SRS146764\_contig\_number\_16680
1262937
1

105
family
2005525

25
genus
375288

SRS014235\_contig\_number\_3394SRS014287\_contig\_number\_16318SRS015663\_contig\_number\_25596SRS019068\_contig\_number\_39628SRS019685\_contig\_number\_8348SRS019968\_contig\_number\_13494SRS023715\_contig\_number\_24427SRS023829\_contig\_number\_contig-100\_321.14744SRS043667\_contig\_number\_5986SRS045244\_contig\_number\_7100SRS046369\_contig\_number\_9902SRS051031\_contig\_number\_576SRS056537\_contig\_number\_41855SRS063370\_contig\_number\_12509SRS075398\_contig\_number\_1433SRS075878\_contig\_number\_4627SRS077294\_contig\_number\_16503SRS078176\_contig\_number\_16412SRS098717\_contig\_number\_3472SRS143085\_contig\_number\_1892SRS147271\_contig\_number\_19061SRS148970\_contig\_number\_contig-100\_39.40SRS893187\_contig\_number\_10154SRS893252\_contig\_number\_13516SRS893382\_contig\_number\_5675
105


SRS018575\_contig\_number\_121
1947048
species
1

1

SRS148721\_contig\_number\_62
2292053
species

species

SRS020622\_contig\_number\_1012
2292363
1

7

SRS017103\_contig\_number\_37036SRS020394\_contig\_number\_15330SRS024331\_contig\_number\_33186SRS053214\_contig\_number\_1525SRS077730\_contig\_number\_13938SRS1041133\_contig\_number\_15354SRS144362\_contig\_number\_38853
469591
species

3
species
2293117

SRS020328\_contig\_number\_32911SRS022713\_contig\_number\_3994SRS057478\_contig\_number\_6493

species

SRS018623\_contig\_number\_16761SRS020233\_contig\_number\_55653SRS075341\_contig\_number\_10973
2293116
3

50

SRS011084\_contig\_number\_1891SRS012273\_contig\_number\_21844SRS013158\_contig\_number\_contig-100\_354.36571SRS015065\_contig\_number\_21714SRS015217\_contig\_number\_29558SRS015782\_contig\_number\_contig-100\_468.240275SRS015794\_contig\_number\_2028SRS015890\_contig\_number\_6710SRS015960\_contig\_number\_13599SRS016267\_contig\_number\_10332SRS016629\_contig\_number\_contig-100\_7.8SRS017307\_contig\_number\_12761SRS018836\_contig\_number\_16392SRS018888\_contig\_number\_2939SRS018984\_contig\_number\_3507SRS019397\_contig\_number\_2481SRS019787\_contig\_number\_10705SRS021948\_contig\_number\_31520SRS022071\_contig\_number\_29722SRS023526\_contig\_number\_26439SRS023583\_contig\_number\_11163SRS043768\_contig\_number\_15340SRS044535\_contig\_number\_15910SRS045004\_contig\_number\_20083SRS045645\_contig\_number\_7558SRS045713\_contig\_number\_16760SRS046712\_contig\_number\_758SRS047044\_contig\_number\_305SRS049712\_contig\_number\_5530SRS051610\_contig\_number\_9684SRS051882\_contig\_number\_3950SRS053398\_contig\_number\_26737SRS054590\_contig\_number\_295SRS056259\_contig\_number\_13639SRS058145\_contig\_number\_1014SRS062701\_contig\_number\_4962SRS101376\_contig\_number\_20987SRS1041091\_contig\_number\_14597SRS1041134\_contig\_number\_5665SRS1041142\_contig\_number\_539SRS1041147\_contig\_number\_3676SRS1055038\_contig\_number\_1539SRS142712\_contig\_number\_2128SRS143876\_contig\_number\_24905SRS144603\_contig\_number\_11850SRS147272\_contig\_number\_639SRS147377\_contig\_number\_3522SRS148511\_contig\_number\_13802SRS893259\_contig\_number\_3172SRS893288\_contig\_number\_652
823
species

3
species
658662

SRS1041132\_contig\_number\_3561SRS1041144\_contig\_number\_6184SRS893230\_contig\_number\_1673

2293114

SRS019161\_contig\_number\_13783SRS075984\_contig\_number\_11763SRS104327\_contig\_number\_2187SRS142542\_contig\_number\_4015SRS148817\_contig\_number\_contig-100\_400.84846
species
5

3
563193

SRS019601\_contig\_number\_5385SRS056273\_contig\_number\_11532SRS075078\_contig\_number\_15635
species

3
46503

SRS104036\_contig\_number\_2678SRS148196\_contig\_number\_37492SRS148253\_contig\_number\_8022
species
